# Supplementary material for: New Insights on the Role of Anti-PD-L1 and Anti-CTLA-4 mAbs on Different Lymphocytes Subpopulations in TNBC
Source: Cancers (Basel). 2022 Oct 27;14(21):5289. doi: 10.3390/cancers14215289 (PMC9656156; doi:10.3390/cancers14215289)
Supplement: Supplementary file 1 [file cancers-14-05289-s001.zip › cancers-1957876-supplementary.pdf]

New Insights on the Role of Anti-PD-L1 and Anti-CTLA-4 mAbs on Different Lymphocytes Subpopulations in TNBC

Rosa Rapuano Lembo [1,2,†], Lorenzo Manna [1,3,†], Guendalina Froechlich [1,2], Emanuele Sasso [1,3], Margherita Passariello [1,3,*] and Claudia De Lorenzo [1,3,*]

1 Ceinge—Biotecnologie Avanzate s.c.a.r.l., via Gaetano Salvatore 486, 80145 Naples, Italy
2 European School of Molecular Medicine, University of Milan, 20122 Milan, Italy
3 Department of Molecular Medicine and Medical Biotechnology, University of Naples "Federico II", Via Pansini 5, 80131 Naples, Italy
* Correspondence: margherita.passariello@unina.it (M.P.); cladelor@unina.it (C.D.L.); Tel.: +39-081-3737868 (C.D.L.)
† These authors contributed equally to this work.

Supplementary Figures and Legends

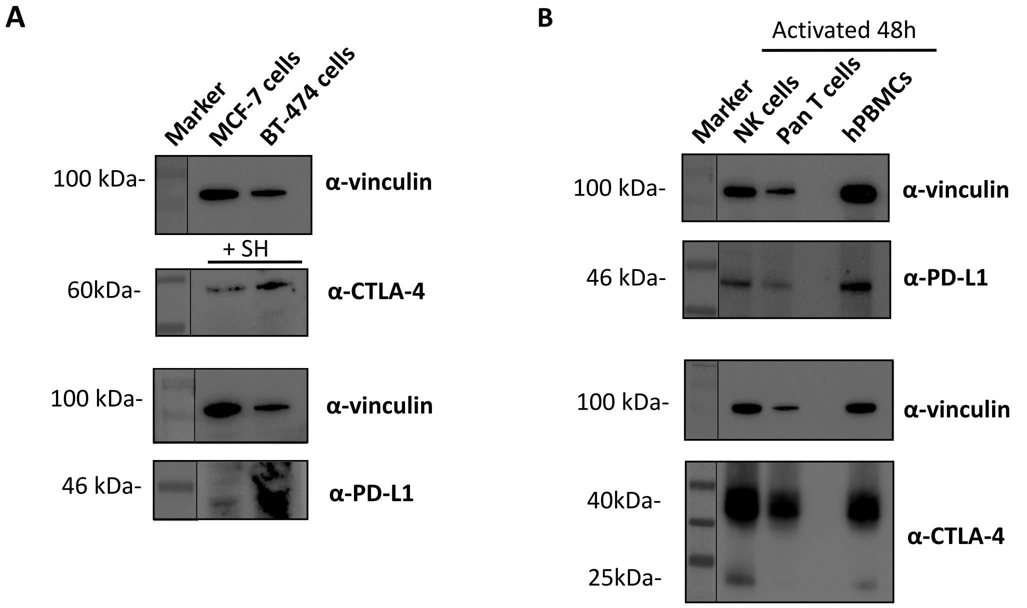

Supplementary Figure S1. Western Blotting analyses to evaluate the expression levels of ICs proteins. (**A**) The extracts from MCF-7 and BT-474 breast tumor cells were analyzed by using the commercial anti-CTLA-4 or anti-PD-L1 mAbs; for CTLA-4 expression the immunoblot was analyzed under reducing (+SH) conditions. (**C-D**) The extracts from NK, Pan T and hPBMCs immune cells, after activation with SEB (50ng/ml) for 48 h, were analyzed by using the commercial anti-PD-L1 or anti-CTLA-4 mAbs. The intensity of the bands corresponding to ICs was normalized to vinculin.

A

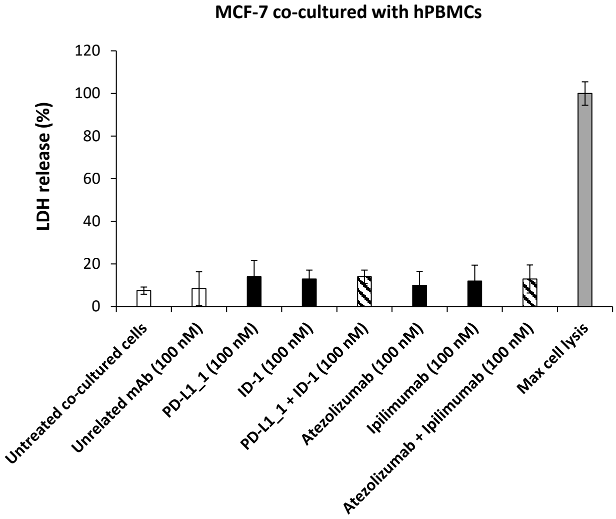

B

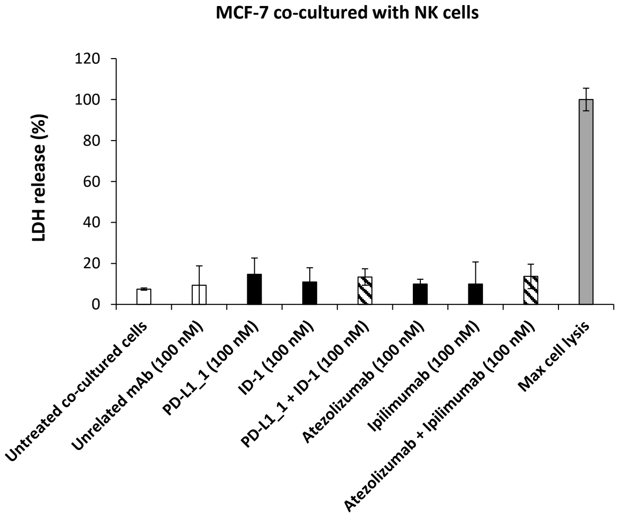

C

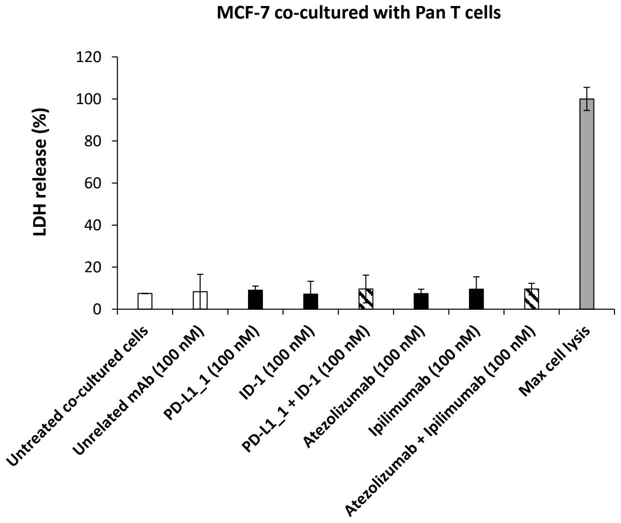

Supplementary Figure S2. LDH assays on MCF-7 breast cancer cells co-cultured with the indicated immune cells in the presence of immunomodulatory mAbs. Cells were cultured with the indicated immune cells in the absence or presence of the novel immunomodulatory mAbs, used alone (black bars) or in combination (striped bars), in comparison with Atezolizumab and Ipilimumab, at the concentrations of 100 nM for 48 h. Untreated cells or cells treated with an unrelated mAb were used as negative controls (white bars). The release of LDH was measured in the supernatants of the cells untreated or treated as indicated, and the tumor cell lysis expressed as previously described. Error bars depict means ± SD.

Full-length blot of Western Blotting analyses

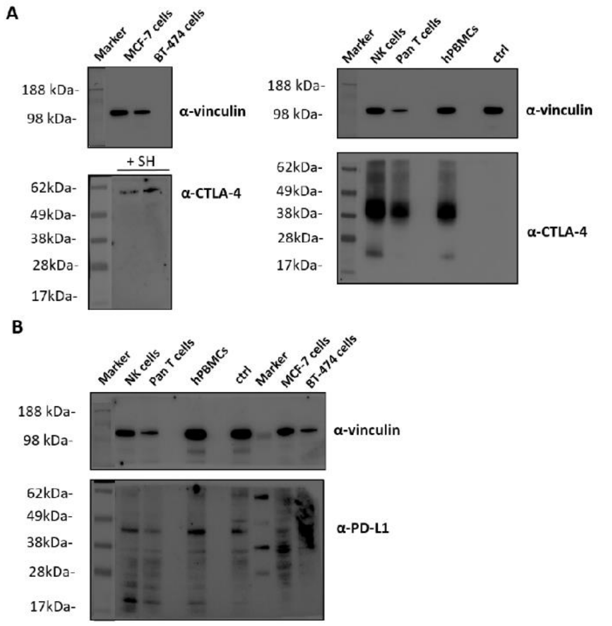

Original Images. Full-length blots of Supplementary **Figure S1**.
